# Identifying local authority need for, and uptake of, school-based physical activity promotion in England–a cluster analysis

**DOI:** 10.1093/pubmed/fdab138

**Published:** 2021-05-04

**Authors:** Tishya Venkatraman, Kate Honeyford, Bina Ram, Esther M F van Sluijs, Céire E Costelloe, Sonia Saxena

**Affiliations:** Department of Primary Care and Public Health, Imperial College London, London, W6 8RP, UK; Department of Primary Care and Public Health, Imperial College London, London, W6 8RP, UK; Department of Primary Care and Public Health, Imperial College London, London, W6 8RP, UK; MRC Epidemiology Unit & Centre for Diet and Activity Research (CEDAR), University of Cambridge, Cambridge, CB2 0QQ, UK; Department of Primary Care and Public Health, Imperial College London, London, W6 8RP, UK; Department of Primary Care and Public Health, Imperial College London, London, W6 8RP, UK

**Keywords:** children, health promotion, physical activity

## Abstract

**Background:**

School-based physical activity interventions such as The Daily Mile (TDM) are widely promoted in children’s physical activity guidance. However, targeting such interventions to areas of greatest need is challenging since determinants vary across geographical areas. Our study aimed to identify local authorities in England with the greatest need to increase children’s physical activity and assess whether TDM reaches school populations in areas with the highest need.

**Methods:**

This was a cross-sectional study using routinely collected data from Public Health England. Datasets on health, census and the built environment were linked. We conducted a hierarchical cluster analysis to group local authorities by ‘need’ and estimated the association between ‘need’ and registration to TDM.

**Results:**

We identified three clusters of high, medium and low need for physical activity interventions in 123 local authorities. Schools in high-need areas were more likely to be registered with TDM (incidence rate ratio 1.25, 95% confidence interval: 1.12–1.39) compared with low-need areas.

**Conclusions:**

Determinants of children’s physical activity cluster geographically across local authorities in England. TDM appears to be an equitable intervention reaching schools in local authorities with the highest needs. Health policy should account for clustering of health determinants to match interventions with populations most in need.

## Introduction

School-based physical activity interventions such as The Daily Mile™ (TDM) are widely promoted as a means to increase children’s physical activity. TDM is a teacher-led school-based active mile intervention, which involves children running or jogging for 15 minutes at their own pace at least three-times a week.^1^ TDM aims to increase children’s physical activity, which has important benefits for children’s health and well-being.^2–4^ TDM is easy to implement,^5^ increases children’s cardiorespiratory fitness,^6–11^ and can contribute to children meeting the physical activity recommendations.^12^ Children’s physical activity across England falls significantly short of the Chief Medical Officer’s recommendations of an average of 60 minutes or more daily.^13^ Only around half of children in England meet this recommendation.^14^^,^^15^ Targeting interventions to increase children’s physical activity to areas of greatest need is challenging since physical activity and obesity rates vary in both children and adults geographically across England.^15–17^

Populations in the South East of England are more active compared with those living in the North.^16^ Reasons for this difference include changing patterns of health behaviours across industrialized areas of England,^16^ stark geographical differences in the built environment and household deprivation that in turn can determine access to safe space for children to be active. Many of the determinants of children’s physical activity are interconnected and spatially clustered across England. For example, children’s physical activity is higher in households where parents are themselves physically active^18^^,^^19^ and in neighbourhoods with access to green space.^20^^,^^21^ Whereas, adult and child obesity and sedentary behaviours are between two and four times higher in areas of social deprivation^22^ and can be partly explained by the cost of healthy foods and association between food prices, deprivation and obesity.^23^ Additionally, good quality green space is more likely to be limited in areas of social deprivation.^24^^,^^25^ In these areas, school-based physical activity interventions may have added value for children who are disadvantaged by lack of space or support for children to be active. Hence, matching public health interventions to the needs of local populations is more efficient and equitable than blanket policies directed at whole populations^26^; and in recent years, there has been a push to consider the accumulation of health risks to plan for health needs.^27^

Previous studies have not reported how children’s need for physical activity is spatially clustered across England and describing this need is important for public health practitioners. Thus, while TDM has been immensely popular with one in five primary schools registered in England,^28^ its ability to address inequalities in children’s physical activity in England rests on whether it can reach schools in areas that could benefit from it the most. As TDM is a grassroots movement, we hypothesized that school staff who are aware of the need to increase physical activity and prevent obesity may be self-identifying populations and therefore have greater uptake. We aimed to identify local authorities in England with the greatest need to increase children’s physical activity and assess whether TDM reaches school populations in areas with the highest need.

## Methods

### Defining need for physical activity promotion

To define local authority area ‘need’ we first conducted a literature review to identify relevant determinants of children’s need for physical activity promotion using data available from sources described below. Our assessment of local authority health ‘need’ included measures of physical activity, excess weight status, mental health, access and utilization of outdoor space for exercise and the proportion of children on free school meals as a proxy measure of deprivation (details of which measures were taken from which sources are available in Table 1). Although there are many determinants that can be used to measure health profiles by area, we were restricted by what was routinely available across England. Data on uptake of TDM was provided by The Daily Mile Foundation.

**Table 1:** County and Unitary Authority characteristics including variable definitions and data sources

*Measure*	*Definition*	*Data source*	*Year*	*Number of local authorities reporting*	*Mean prevalence across local authorities (SD)*
Children’s physical activity	Percent of children in a local authority aged 5–16 years that meet the UK Chief Medical Officers’ recommendations for physical activity (an average of at least 60-minute moderate-vigorous intensity activity per day across the week).	Active Lives Children and Young People Survey, Sport England	2018–19	131	45.3(6.4)
Adult physical activity	Percent of respondents aged 19 and over, with valid responses to questions on physical activity, doing at least 150 moderate intensity equivalent minutes physical activity per week in bouts of 10 minutes or more in the previous 28 days.	Active Lives Adult Survey, Sport England	2018–19	149	65.6 (5.4)
Adolescent sedentary time	Percentage with a mean daily sedentary time in the last week over 7 hours per day at age 15.	What About YOUth survey	2014–15	147	70.7 (4.3)
Excess weight status at age 5–6	Reception: percent of overweight (including obese).	National Child Measurement Programme	2018–19	147	22.8 (2.7)
Excess weight status at age 11–12	Year 6: percent of overweight (including obese).	National Child Measurement Programme	2018–19	147	34.9 (4.5)
Adult excess weight status	Percentage of adults (aged 18+) classified as overweight or obese.	Active Lives Adult Survey, Sport England	2018–19	149	62.1 (6.4)
Free school meals: % uptake among all pupils	Percent of pupils known to be eligible for and claiming free school meals who attend a state funded nursery, primary, secondary or a special school.	Department for Education School Census	2017–18	152	14.3 (5.6)
School pupils with social, emotional and mental health needs: % of school pupils with social, emotional and mental health needs	The number of school children who are identified as having social, emotional and mental health needs expressed as a percentage of all school pupils.	Department for Education special educational needs statistics	2017–18	151	2.5 (0.6)
Utilization of outdoor space for exercise/health reasons	The proportion of residents in each area taking a visit to the natural environment for health or exercise purposes. Visits to the natural environment are defined as time spent ‘out of doors’ e.g. in open spaces in and around towns and cities, including parks, canals and nature areas; the coast and beaches and the countryside including farmland, woodland, hills and rivers. This could be anything from a few minutes to all day. It may include time spent close to home or workplace, further afield or while on holiday in England.	Natural England: Monitor of Engagement with the Natural Environment survey	2015–16	138	17.7 (4.1)

### Study design and data sources

This was a cross-sectional study. We used publicly available data on multiple health indicators extracted from Public Health England^29^ for all 152 upper-tier local authorities in England. These include metropolitan districts, London boroughs, unitary authorities and county councils. Data that are available and were used for our study are from Active Lives Surveys, National Child Measurement Programme, Department for Education Special Education Needs Statistics, Natural England: Monitor of Engagement with the Natural Environment Survey and Department for Education School Census (more detail on the data sources is presented in Supplementary Material A1). Where available, we used the most recent data for each data source; this was not the same year for each data source.

### Statistical analysis

Only local authorities with complete data for all health behaviours and demographic variables were included in the cluster analysis. Local authorities with missing data were analysed for patterns of missingness and a sensitivity analysis was run to explore differences by health behaviours in excluded versus included data (Supplementary Material A2).

We ran a cluster analysis to enable grouping of observations, in this case local authorities, based upon similarity across the set of characteristics defined above (‘need’). We standardized (converted to Z-scores) all the variables before any analysis to minimize the influence of different measurement scales of each of the variables. To determine the most appropriate clustering method, we used the R package clValid.^30^ This allows simultaneous comparison of multiple clustering algorithms such as hierarchical (divisive and agglomerative), k-means and partition around medoids to identify the best clustering approach and the optimal number of clusters. In addition, we used the R package NbClust to further determine the optimum number of clusters.^31^ This package uses 30 indices for choosing the best number of clusters for the data.

We assessed internal measures to determine the quality of the cluster solution by calculating the silhouette coefficient indicating cohesion and separation. The silhouette ranges from −1 to +1; a high value indicates that the object is well matched to its own cluster and poorly matched to neighbouring clusters. We also calculated stability measures, specifically, the average proportion of non-overlap (APN), which measures the average proportion of observations not placed in the same cluster by clustering based on the full data and clustering based on the data with a single column removed and ranges from 0 to 1. For a good clustering algorithm, we would expect the APN value to be small.^32^ We used Ward’s linkage method as it identifies the strongest clustering in our data. Ward’s linkage reduces the variance in each cluster and maximizes the homogeneity; this means that the local authorities have similar interpretable profiles. To examine and confirm cluster profiles and differences between health behaviours, we used analysis of variance (ANOVA) and tested for multiplicity using Tukey’s test. The coefficient of variation was also calculated to present a normalized measure of the variation in variables to help assess their weight in cluster formation. Using the cluster membership derived from the cluster analysis, real value means and standard deviations (SDs) for each variable were derived.

We estimated the association between the ‘need’ for physical activity interventions in local authorities and uptake to TDM in the clusters derived from the hierarchical cluster analysis with incidence rate ratios (IRR) and their 95% confidence intervals (CIs) via a Poisson regression. All statistical analyses were done using R software version 3.5.2 (20-12-2018).

## Results

### Study population

We identified complete data for 123 of 152 (81%) local authorities across England Table 1.

We did not find any differences between included and excluded local authorities due to missing data (Supplementary Material A2).

### Cluster formation and definition

After evaluating the internal and stability measures of different clustering methods, we used a hierarchical agglomerative clustering algorithm (bottom-up) to identify subgroups within the data. The APN favoured a hierarchical cluster analysis with the optimum number of clusters being 3 (APN = 0.04). The silhouette coefficient for the hierarchical cluster analysis using three clusters was 0.21 in the total sample, indicating a fair model. We confirmed that three clusters were optimum by using the clValid package. This selected either two or three as the preferred number of clusters, with a slight preference for 2 (10 measures identified two as the optimum and 8 identified three clusters). We selected three as it was the most interpretable (more information provided in Supplementary Material A3).

### Cluster profiles

**Fig. 1:**
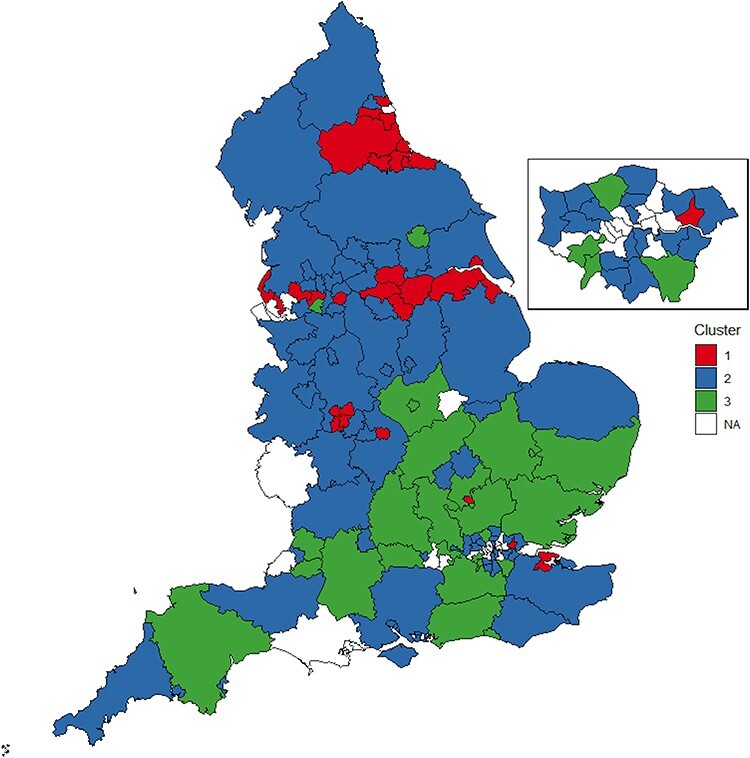
Map showing the geographic distribution of need identified. Cluster 1: high-need local authorities; cluster 2: medium-need local authorities; cluster 3: low-need local authorities; NA = missing.

Three clusters were generated from the hierarchical cluster analysis. Cluster 1 had 30 local authorities (24.4% of 123 local authorities), cluster 2 had 69 (56.1% of 123 local authorities) and cluster 3 had 24 (19.5% of 123 local authorities) (Fig. 1; Supplementary Material A4 for list of local authorities by cluster). Variables with greater variation are more important in cluster formation as they allow for higher discrimination between clusters. Coefficient of variation values were the highest among children’s indicators suggesting that they were most important in cluster formation (Table 2). Post hoc assessment of differences between clusters showed significant differences in all bar two need indicators of need.

**Table 2:** Cluster outputs and characteristics of counties and unitary authorities according to the need for physical activity interventions assessed

*Clusters*	*High-need areas* (N = 30) *Cluster* 1	*Medium-need areas* (N = 69) *Cluster* 2	*Low-need areas* (N = 24) *Cluster* 3	*Coefficient of variation*
**Local authority indicators of health need**	**% (SD)**	**% (SD**	**% (SD)**	
Excess weight status at age 5–6 ^*^	25.2 (1.2)	22.9 (1.9)	19.5 (1.9)	11.6
Excess weight status at age 11–12^*^	38.2 (3.0)	35.4 (3.3)	28.6 (2.6)	12.9
Children physical activity^*^	42.8 (6.5)	44.6 (5.8)	49.0 (6.1)	14.1
Children on free school meals^*^	17.6 (4.0)	14.3 (4.6)	8.4 (1.8)	36.3
School pupils with social, emotional and mental health needs	2.5 (0.5)	2.4 (0.6)	2.3 (0.5)	22.9
Adolescent sedentary time^*^	75.5 (1.2)	70.2 (2.9)	66.4 (3.8)	6.0
Adult excess weight^*^	68.2 (3.2)	61.9 (4.7)	57.9 (4.6)	8.9
Adult physical activity^*^	60.2 (2.9)	66.1 (4.5)	69.6 (4.5)	8.1
Use of outdoor space	17.8 (4.6)	17.0 (3.2)	18.6 (4.6)	22.2

*N* = number of local authorities.

^*^Differences between clusters were observed by ANOVA. All three clusters are significantly different at *P* < 0.001 (Tukey post hoc).

Local authorities in cluster 1, described as ‘high-need’ areas displayed the poorest health outcomes overall, apart from the utilization of outdoor space for exercise and health reasons, compared with local authorities in the medium- and low-need clusters and comprised of 30 local authorities. A total of 38% of children in these local authorities were overweight or obese at the end of primary school, and 42% of children were meeting the recommended levels of physical activity. Further, there were more than double the proportion of children on free school meals in this cluster compared with those in the healthiest ‘low-need’ cluster and almost 70% of adults in these local authorities were overweight or obese. These local authorities are concentrated in certain areas such as the West Midlands, South Yorkshire and the North East of England (Fig. 1).

Cluster 2 described as ‘medium-need’ areas comprised of 69 local authorities. The local authorities in the medium-need cluster had moderate values for all health behaviours except for the utilization of outdoor space for exercise and health reasons. Medium-need local authorities were spread across England but were more concentrated in north and central England.

Cluster 3 described as ‘low-need’ areas comprised of 24 local authorities that had the best health outcomes across all health behaviours. It had the least deprived children and had the highest utilization of outdoor space. In total, 57% of the adult population was either overweight or obese, 13% less than adults in the high-need local authorities, and less than half of children met the recommended levels of physical activity. These local authorities comprised of some of the most affluent boroughs of London, a large proportion of the East of England, affluent areas of South West England (e.g. Bath) and parts of South East England (e.g. Buckinghamshire).

### TDM uptake associated with ‘need’

Highest uptake for TDM was in schools in local authorities defined as high need. These schools were 25% more likely to register to take part in TDM (IRR 1.25 [95% CI −1.12, 1.39] *P* < 0.001) compared with those in areas with low need (Table 3).

**Table 3:** IRRs of uptake to TDM by clusters

*Cluster*	*N and % of registered TDM schools*	*IRR* (95% CI)	P *value*
Cluster 1: high need	530 (25.09%)	1.25 (1.12, 1.39)	<0.001
Cluster 2: medium need	1841 (21.48%)	1.07 (0.98, 1.16)	0.127
Cluster 3: low need	755 (20.11%)	Reference	

## Discussion

### Main findings of this study

We identified three distinct clusters of high, medium and low need for physical activity interventions in 123 local authorities representing ~4.5 million primary school children in England. The three clusters of need were characterized by determinants of children’s health need for physical activity promotion. The important drivers of need included deprivation in children, children’s social emotional and mental health needs, children’s physical activity and use of outdoor space for exercise and health reasons. School populations located in the highest need cluster for physical activity interventions were 25% more likely to be registered to TDM than schools in the lowest need cluster; where registration ranged from one in five schools in the low-need cluster to one in four in the high-need cluster. This affirms current physical activity and obesity policy in England that simple school-based interventions can be effective and address health inequalities.

### What is already known on this topic

Our findings showed that local authorities with low physical activity, high sedentary behaviour, high excess weight and high deprivation were clustered together. This is consistent with previous studies where patterns of high physical activity are significantly associated with parental education^33^^,^^34^ and parental income.^35^^,^^36^ Further, we found that local authorities with the poorest health outcomes (high-need cluster) were concentrated in northern England and those with the best health outcomes were concentrated in the south (low-need cluster). A 2011 study^37^ found similar clustering patterns when identifying local authorities with multiple health and social needs—where membership of the worst cluster was focussed in the North of England, and the local authorities with the best health outcomes were in the East and South East. They reported that people in local authorities in the cluster with the worst health outcomes face a lifelong public health deficit across numerous health outcomes including healthy eating habits, physical activity in children and ultimately reduced life expectancy. Although some behaviours have improved, our study indicates that little has changed over the last 10 years^37^ with respect to clustering of the health behaviours we analysed and how they are distributed across the country. This attests to the urgent need to level up the geographical disparities observed and implement targeted interventions that reduce inequalities in children’s physical activity and health.

A recent study in Wales that examined TDM in schools found that it improved fitness equally among children of varying deprivation groups.^6^ Our study reports that schools in high-need local authorities were more likely to register to TDM compared with schools in low-need areas, implying that schools that register to TDM may have the potential to reduce some inequalities observed in children’s physical activity in the UK.^38^

Similar results were observed in a study in Canada; after implementing a school-based physical activity intervention in disadvantaged neighbourhoods,^39^ physical activity levels of children in disadvantaged neighbourhoods approximated those in wealthier neighbourhoods and the intervention reached high-need groups such as children with low activity and those that were overweight. Further, while it is reassuring to know that TDM is reaching high-need areas, there is an urgent need to increase children’s physical activity levels across England given we found almost 50% of children in low-need areas were not meeting the physical activity recommendations.

A potential explanation for the increased uptake of TDM in high-need local authorities could be that it is a grassroots movement and as TDM is teacher led, teachers possibly self-identify children in their schools as populations that could benefit from TDM. Further, the UK government’s mention of TDM in its child obesity strategy^40^ may have resulted in some local authorities actively promoting it. TDM is a ready-made package that schools can spend their Sports Premium resources on, which could be driving uptake. Widespread adoption (95% of pupils) of TDM has been observed in the short term in one school in England.^41^ However, for long-term physical activity adherence in children, intrinsic motivation is vital.^42^^,^^43^ While it is possible that TDM can increase physical activity in children^7^^,^^8^ in an equitable manner in the short term, it must be coupled with other sustainable behaviour change interventions that facilitate this intrinsic motivation, which includes enjoyment and inherent satisfaction of physical activity.

### What this study adds

Our use of a clustering design advances on previous research by accounting for the interaction of several determinants of children’s physical activity. Further, this was the first study to examine how health behaviours and built environment features are clustered and spatially distributed across England by using data representativeness of the whole population. Visualizing the determinants of children’s physical activity distinctly spatially clustered across local authorities in our study is a powerful tool for illustrating their coexistence. This study provides a robust methodology to evaluate interventions with the awareness of the interactions between the underlying determinants these interventions aim to target. The identification of clusters of local authorities that share patterns of need for physical activity interventions may help to guide public health and other policy interventions. For example, successful interventions in one local authority might transfer more easily to other local authorities within the same cluster, as these areas may share similar challenges and contextual features. Our study helps to understand the geographic variation of health needs and finetuning interventions to target combinations of the underlying health behaviours that give rise to the clusters seen in this study and previously, instead of targeting single behaviours and determinants of children’s physical activity in isolation.^37^

While our study suggests that schools in areas with the greatest need register to TDM, it is imperative to study implementation over time given that previous studies have found teacher buy-in to be crucial for long-term adherence to school-based running programmes.^5^^,^^44^ It would also be of interest to include additional determinants of children’s physical activity such as policy and behavioural determinants that are currently unavailable in routine data, to be able to understand the bigger upstream drivers of need that are important.^45^

### Limitations of this study

The public health metrics used in this study were identified and quality assured by Public Health England. However, some of the data were missing, particularly for local authorities in London. Further self-reported data such as physical activity prevalence was used for this study and is subject to recall bias.

Data on uptake to TDM was obtained via TDM Foundation and is based on whether a school officially registered to their website. Whether schools actually implement TDM has not been validated. School registration to TDM may not equate to effective implementation across the school. Moreover, schools not officially registered may have adopted another active mile initiative or other activities similar to TDM.

We used upper-tier local authorities in this study to make the results useful for policymakers at the national and local authority level. However, this means that the cluster solution found could be influenced by variation in types of local authority and within local authorities. There may also be variation at a more granular level (lower-tier local authorities). Thus, using smaller areas may remove some of this variation and allow for the detection of smaller differences. Finally, this was a cross-sectional study and can only identify a snapshot of clustering of health behaviours and its association with TDM registration. Longitudinal studies would determine the changing health needs of the population and help examine implementation of TDM over time. Although there are limitations, we have clearly shown that health policy should account for clustering of health determinants to match interventions with populations most in need.

## Supplementary Material

Additional_File_1_fdab138

Additional_File_2_fdab138

Additional_File_3_fdab138

Additional_File_4_fdab138

## Data Availability

All data from the Active Lives Surveys, National Child Measurement Programme, Department for Education Special Education Needs Statistics, Natural England: Monitor of Engagement with the Natural Environment survey and Department for Education School Census are publicly available from Public Health England Fingertips at https://fingertips.phe.org.uk/ Data on school registration to The Daily Mile was obtained from The Daily Mile Foundation and is not publicly available. For access to this data, requests must be directed to The Daily Mile Foundation.

## Notes

**Tishya Venkatraman**, PhD Student

**Kate Honeyford**, Research Associate

**Bina Ram**, Research Associate

**Esther M.F. van Sluijs**, MRC Programme Leader

**Céire E. Costelloe**, Senior Lecturer and Director Global Digital Health Unit

**Sonia Saxena**, Professor of Primary Care
