# Supplementary material for: Identifying local authority need for, and uptake of, school-based physical activity promotion in England–a cluster analysis
Source: J Public Health (Oxf). 2021 May 4;44(3):694–703. doi: 10.1093/pubmed/fdab138 (PMC9424056; doi:10.1093/pubmed/fdab138)
Supplement: Additional_File_1_fdab138 [file additional_file_1_fdab138.docx]

## **Additional File 1**

**Data sources**

*Active Lives Survey for Children and Young People*

The Active Lives Survey for Children and Young People is a schools-based survey that collects self-reported physical activity data from children across more approximately 2,000 schools in England. The survey uses a 7-day reference period to record the activity children had done on each day. The estimates are weighted to be representative of the national population.(1,2)

#### Active Lives Adult Survey

The Active Lives Adult Survey includes data on adult self-reported physical activity, height and weight. This survey uses a 28-day reference period for adults to record the number of minutes of physical activity (of at least 10 minutes) and then divides the number of minutes by four to calculate a weekly average. The data obtained from the survey are weighted to be representative of the whole population at each level of geography. Weight status data from the survey has also been age-standardised to improve comparability of excess weight prevalence between local authorities. The estimates are weighted to be representative of the national population.(3,4)

#### What About YOUth survey

The What About YOUth survey has local level data on a range of topics relating to young people, including emotional wellbeing, diet, and physical activity. The survey randomly selected around 300,000 15-year-olds from the Department for Education's National Pupil Database, which contains details of every pupil in England. They had around 120,000 completed questionnaires being returned. The estimates are weighted to be representative of the national population.(5)

#### National Child Measurement Programme

We defined children’s excess weight status at two time points in our study – once at the beginning of primary school (children aged 5-6 years) and once at the end (children aged 11-12 years). We defined excess weight status as the percent of children classified as overweight or obese in a local authority. Children are classified as overweight (including obese) if their BMI is on or above the 85th centile of the British 1990 growth reference (UK90) according to age and sex. This data was extracted from the National Child Measurement Programme; a nationally mandated surveillance programme that that collects height and weights (objectively measured) converted to weight status for over 1 million children (98% of all children) entering and leaving primary school in England each year.(6)

#### Department for Education

The School Census collects aggregate information annually from all state funded schools and includes data on more than 15,000 schools and over 4.5 million children,(7) including pupil numbers, ethnicity, or if they are disadvantaged. The National Pupil Database includes pooled data, based on multiple data collections on pupils aged 3-19 years in state funded schools in England. It contains data on pupils’ educational attainment from national annual standardised assessments conducted each academic year, and whether a child has any special educational needs.(8)

*Natural England: Monitor of Engagement with the Natural Environment survey*

The Monitor of Engagement with the Natural Environment survey collects data on engagement with the natural environment (all green open spaces in and around towns and cities as well as the wider countryside and coastline), with a focus on visits to the natural environment. During each survey interview, respondents are asked to indicate how many visits they have taken to the natural environment in the last 7 days. They survey includes data from approximately 45,000 respondents. The estimates are weighted to be representative of the national population.(9)

**References:**

1. Active Lives Children and Young People Survey 2018/19 | Ipsos MORI [Internet]. [cited 2020 May 14]. Available from: https://www.ipsos.com/ipsos-mori/en-uk/active-lives-children-and-young-people-survey-201819

2. Sport England. The Active Lives: Children And Young People Survey – Questionnaire Content.

3. Sport England. Method behind Active Lives [Internet]. [cited 2020 May 14]. Available from: https://www.sportengland.org/know-your-audience/data/active-lives#method_behind_active_lives

4. Sport England. Active Lives Adult Survey November 2018/19 Report. 2020 Apr.

5. NHS Digital. Health and Wellbeing of 15-year-olds in England - Main findings from the What About YOUth? Survey 2014 [Internet]. [cited 2020 May 14]. Available from: https://digital.nhs.uk/data-and-information/publications/statistical/health-and-wellbeing-of-15-year-olds-in-england/main-findings---2014

6. Public Health England. National child measurement programme: operational guidance - GOV.UK [Internet]. 2019 [cited 2020 Mar 18]. Available from: https://assets.publishing.service.gov.uk/government/uploads/system/uploads/attachment_data/file/817417/NCMP_Operational_Guidance_2019.pdf

7. Department for Education. School census 2018 to 2019: guide for schools and LAs - GOV.UK [Internet]. 2019 Feb [cited 2020 Mar 12]. Available from: https://www.gov.uk/government/publications/school-census-2018-to-2019-guide-for-schools-and-las

8. Jay MA, Mc Grath-Lone L, Gilbert R. Data Resource: the National Pupil Database (NPD). Int J Popul Data Sci. 2019 Mar 20;4(1).

9. Natural England. Monitor of Engagement with the Natural Environment The national survey on people and the natural environment [Internet]. 2018 [cited 2020 May 29]. Available from: https://www.gov.uk/government/statistics/monitor-of-engagement-with-the-natural-environment-2017-to-2018.
