# Supplementary material for: Identifying local authority need for, and uptake of, school-based physical activity promotion in England–a cluster analysis
Source: J Public Health (Oxf). 2021 May 4;44(3):694–703. doi: 10.1093/pubmed/fdab138 (PMC9424056; doi:10.1093/pubmed/fdab138)
Supplement: Additional_File_2_fdab138 [file additional_file_2_fdab138.docx]

## **Additional File 2**

### Missing data

| **Local authority name** | **Excess Weight Status at Age 5-6 (%)** | **Children Physical Activity (%)** | **Excess Weight Status at Age 11/12 (%)** | **Adult Physical Activity (%)** | **Adolescent Sedentary Time (%)** | **Use of Outdoor Space (%)** | **Adult Excess Weight (%)** | **Children on Free School Meals (%)** | **School pupils with social, emotional and mental health needs (%)** |
| --- | --- | --- | --- | --- | --- | --- | --- | --- | --- |
| **Blackpool** | 28.58 | 59.59 | 38.58 | 54.36 | 79.9 | NA | 66.51 | 25.26 | 3.08 |
| **Bracknell Forest** | 19.44 | NA | 28.39 | 73.87 | 68.6 | NA | 62.52 | 6.64 | 1.9 |
| **Camden** | 20.69 | NA | 36.4 | 71.65 | 68.1 | 17.11 | 46.47 | 24.96 | 2.67 |
| **City of London** | NA | NA | NA | 73.17 | NA | NA | 45.43 | 11.27 | 4.23 |
| **Hackney** | 24.01 | 42.17 | 40.24 | 69.95 | 70.7 | NA | 49.21 | 27.73 | 3.38 |
| **Halton** | 26.64 | NA | 40.31 | 62.85 | 76.9 | 17.55 | 74.41 | 26 | 3.06 |
| **Hammersmith and Fulham** | 19.06 | NA | 35.8 | 68.25 | 64.6 | 17.42 | 48.98 | 20.73 | 2.13 |
| **Herefordshire** | 23.65 | 50.08 | 34.69 | 66.81 | 65.1 | NA | 64.51 | 8.22 | 2.9 |
| **Hounslow** | 23.21 | NA | 38.93 | 63 | 70 | 18.34 | 60.44 | 14.59 | 3.26 |
| **Isles of Scilly** | NA | NA | NA | 78.62 | NA | NA | 63.51 | 4.43 | NA |
| **Kensington and Chelsea** | 19.3 | 45.87 | 35.35 | 72.79 | 69.6 | NA | 49.97 | 18.9 | 2.13 |
| **Lewisham** | 21.31 | NA | 37.3 | 71.63 | 73.4 | 17.69 | 55.6 | 16.14 | 1.98 |
| **Liverpool** | 27.77 | NA | 39.53 | 66.42 | 78.1 | 17.36 | 62.45 | 24.02 | 2.99 |
| **NA** | NA | NA | NA | NA | NA | NA | NA | 10.74 | 2.72 |
| **NA** | NA | NA | NA | NA | NA | NA | NA | 9.8 | 3.11 |
| **NA** | NA | NA | NA | NA | NA | NA | NA | 12.74 | 2.48 |
| **Newham** | 23.73 | NA | 42.66 | 53.12 | 69.9 | 18.82 | 62.97 | 13.58 | 2.03 |
| **North Somerset** | 24.8 | 47.06 | 26.86 | 71.11 | 65.7 | NA | 66.41 | 8.08 | 2.32 |
| **Rutland** | 22.81 | 56.25 | 29.85 | 68.12 | 68.7 | NA | 62.94 | 4.69 | 1.41 |
| **South Tyneside** | 24.66 | 46.98 | 38.13 | 60.27 | 77.8 | NA | 71.66 | 19.17 | 2.72 |
| **Southend-on-Sea** | 22.41 | NA | 33.23 | 62.97 | 73.9 | 15.11 | 67.98 | 11.63 | 2.08 |
| **St. Helens** | 28.16 | NA | 36.63 | 61.75 | 78.3 | 21.2 | 72.55 | 15.78 | 2.77 |
| **Thurrock** | 22.31 | NA | 37.9 | 60.91 | 76 | 36.9 | 68.97 | 11.43 | 2.33 |
| **Tower Hamlets** | 21.44 | NA | 41.49 | 67.19 | 67.6 | 15.7 | 49.11 | 33.58 | 3.02 |
| **Waltham Forest** | 21.75 | NA | 38.01 | 63.01 | 69.5 | 14.33 | 54.49 | 13.96 | 2.6 |
| **Wandsworth** | 17.46 | NA | 32.87 | 72.29 | 64.5 | 19.76 | 55.22 | 15.43 | 3.62 |
| **Westminster** | 21.75 | NA | 37.97 | 68.83 | 66 | 20.07 | 47.98 | 21.99 | 3.19 |
| **Wirral** | 24.05 | NA | 34.04 | 62.1 | 69.8 | 23.39 | 62.48 | 16.95 | 3.55 |
| **Wokingham** | 18.75 | 45.27 | 25.85 | 73.49 | 62.9 | NA | 50.94 | 5.3 | 1.91 |

### Sensitivity Analysis

| **Parameter (%)** | **Included local authorities with complete data Mean (SD)** | **Excluded local authorities due to missing data Mean (SD)** |
| --- | --- | --- |
| Excess Weight Status at Age 5-6 | 22.79 (2.69) | 22.82 (3.00) |
| Excess Weight Status at Age 11-12 | 34.93 (4.48) | 35.88 (4.48) |
| Children Physical Activity Prevalence | 45.26 (6.38) | 49.16 (5.90) |
| Children using Free School Meals | 14.27 (5.63) | 15.65 (7.53) |
| Children’s mental health needs | 2.47 (0.58) | 2.70 (0.63) |
| Adolescent Sedentary Time | 70.73 (4.34) | 70.65 (5.00) |
| Adult Excess Weight Prevalence | 62.08 (6.35) | 59.37 (8.81) |
| Adult Physical Activity Prevalence | 65.62 (5.44) | 66.87 (6.17) |
| Use of Outdoor Space | 17.72 (4.10) | 19.38 (5.38) |
