# Supplementary material for: Identifying local authority need for, and uptake of, school-based physical activity promotion in England–a cluster analysis
Source: J Public Health (Oxf). 2021 May 4;44(3):694–703. doi: 10.1093/pubmed/fdab138 (PMC9424056; doi:10.1093/pubmed/fdab138)
Supplement: Additional_File_3_fdab138 [file additional_file_3_fdab138.docx]

## **Additional File 3**

### Detailed information on Cluster formation and definition

Choosing the best clustering algorithm

Through the clValid package, we assessed internal measures to determine the quality of the cluster solution by calculating the silhouette coefficient indicating cohesion and separation. The silhouette ranges from − 1 to + 1; a high value indicates that the object is well matched to its own cluster and poorly matched to neighbouring clusters. We also calculated stability measures, specifically, the average proportion of non-overlap, which measures the average proportion of observations not placed in the same cluster by clustering based on the full data and clustering based on the data with a single column removed and ranges from 0 to 1. For a good clustering algorithm, we would expect this values to be small.[35] After evaluating the internal and stability measures of different clustering methods, we used a hierarchical agglomerative clustering algorithm (bottom-up) to identify subgroups within the data. The average proportion of non-overlap favoured a hierarchical cluster analysis with the optimum number of clusters being 3, where the value was small = 0.04. The silhouette coefficient for the hierarchical cluster analysis using 3 clusters was 0.21 in the total sample, indicating a fair model.

The hierarchical agglomerative algorithm computes the Euclidean distance between each local authority based on average scores of the need variables we identified. It identifies the two local authorities that have the lowest distance score and links them in a cluster and repeats this process until one cluster remains containing all the local authorities. To measure the dissimilarity between two clusters of observations, we tried different approaches (average, centroid, Ward, and complete). Ward’s linkage method identified the strongest clustering structure in our data. This clustering approach computes the sum of squared distances within the clusters and merges them to minimize it. This process of agglomeration leads to a reduction in the variance of each resulting cluster and maximizes the within-group homogeneity.^[[1]](#footnote-1)^

Determining the optimum number of cluster

The R package clValid indicated 3 clusters being the optimum number for our data. Using NbClust to determine the optimum number of clusters for our data, 10 indices proposed 2 as the best number of clusters, and 8 proposed 3 as the best number of clusters (Figure 1a). The Hubert index is a graphical method of determining the number of clusters. In the plot of Hubert index, we seek a significant knee that corresponds to a significant increase of the value of the measure i.e. the significant peak in Hubert index second differences plot. The D index is a graphical method of determining the number of clusters. In the plot of D index, we seek a significant knee (the significant peak in D index second differences plot) that corresponds to a significant increase of the value of the measure (Figure 1b). There is no definitive answer to choosing the optimum number of clusters since it is an exploratory approach. The resulting cluster solution must be grounded in theory but is also context-dependent. We chose a pragmatic approach to selecting the optimum number of clusters based on the results of multiple indices, but also found that choosing 3 clusters was more informative for our data than 2.

| **Figure S1a**: NbClust’s assessment of the optimum number of clusters for the data  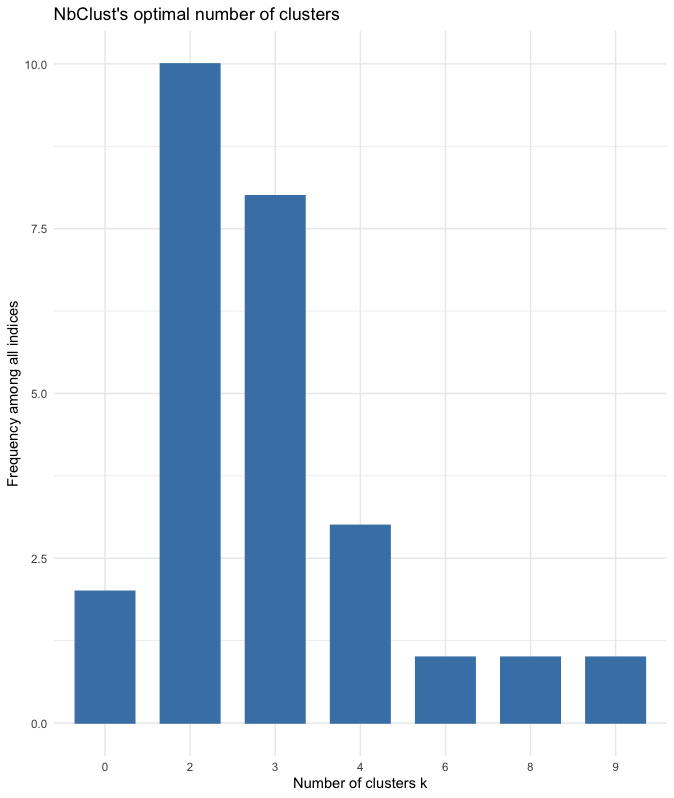 | **Figure S1b:** Hubert index and D index to determine the optimum number of clusters  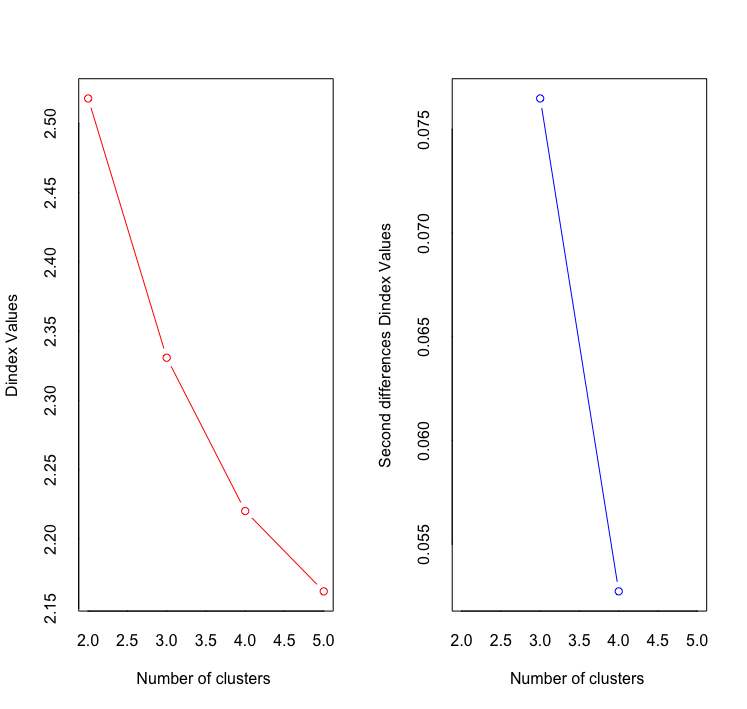 |
| --- | --- |

1. Low LL, Yan S, Kwan YH, *et al.* Assessing the validity of a data driven segmentation approach: A 4 year longitudinal study of healthcare utilization and mortality. *PLoS One* 2018;**13**:e0195243. doi:10.1371/journal.pone.0195243 [↑](#footnote-ref-1)
