# Supplementary material for: Identifying local authority need for, and uptake of, school-based physical activity promotion in England–a cluster analysis
Source: J Public Health (Oxf). 2021 May 4;44(3):694–703. doi: 10.1093/pubmed/fdab138 (PMC9424056; doi:10.1093/pubmed/fdab138)
Supplement: Additional_File_4_fdab138 [file additional_file_4_fdab138.docx]

## **Additional File 4**

### Clusters and local authorities

| **Highest need areas (Cluster 1)** | **Medium-need areas (Cluster 2)** | **Lowest need areas (Cluster 3)** |
| --- | --- | --- |
| Barking and Dagenham | Bedford | Barnet |
| Barnsley | Bexley | Bath and North East Somerset |
| County Durham | Birmingham | Brighton and Hove |
| Coventry | Blackburn with Darwen | Bromley |
| Darlington | Bolton | Buckinghamshire |
| Doncaster | Bradford | Cambridgeshire |
| Dudley | Brent | Central Bedfordshire |
| Gateshead | Bristol | Devon |
| Hartlepool | Bury | Essex |
| Kingston upon Hull | Calderdale | Hertfordshire |
| Knowsley | Cheshire East | Kingston upon Thames |
| Luton | Cheshire West and Chester | Leicestershire |
| Medway | Cornwall | Northamptonshire |
| Middlesbrough | Croydon | Oxfordshire |
| North East Lincolnshire | Cumbria | Richmond upon Thames |
| North Lincolnshire | Derby | South Gloucestershire |
| North Tyneside | Derbyshire | Suffolk |
| Redcar and Cleveland | Ealing | Surrey |
| Rotherham | East Riding of Yorkshire | Trafford |
| Salford | East Sussex | West Berkshire |
| Sandwell | Enfield | West Sussex |
| Sefton | Gloucestershire | Wiltshire |
| Stockton-on-Tees | Greenwich | Windsor and Maidenhead |
| Stoke-on-Trent | Hampshire | York |
| Sunderland | Haringey |  |
| Tameside | Harrow |  |
| Wakefield | Havering |  |
| Walsall | Hillingdon |  |
| Wigan | Isle of Wight |  |
| Wolverhampton | Islington |  |
|  | Kent |  |
|  | Kirklees |  |
|  | Lambeth |  |
|  | Lancashire |  |
|  | Leeds |  |
|  | Leicester |  |
|  | Lincolnshire |  |
|  | Manchester |  |
|  | Merton |  |
|  | Milton Keynes |  |
|  | Newcastle upon Tyne |  |
|  | Norfolk |  |
|  | North Yorkshire |  |
|  | Northumberland |  |
|  | Nottingham |  |
|  | Nottinghamshire |  |
|  | Oldham |  |
|  | Peterborough |  |
|  | Plymouth |  |
|  | Portsmouth |  |
|  | Reading |  |
|  | Redbridge |  |
|  | Rochdale |  |
|  | Sheffield |  |
|  | Shropshire |  |
|  | Slough |  |
|  | Solihull |  |
|  | Somerset |  |
|  | Southampton |  |
|  | Southwark |  |
|  | Staffordshire |  |
|  | Stockport |  |
|  | Sutton |  |
|  | Swindon |  |
|  | Telford and Wrekin |  |
|  | Torbay |  |
|  | Warrington |  |
|  | Warwickshire |  |
|  | Worcestershire |  |
